# Engineering a Polyspecific Pyrrolysyl-tRNA Synthetase by a High Throughput FACS Screen

**DOI:** 10.1038/s41598-019-48357-0

**Published:** 2019-08-19

**Authors:** Adrian Hohl, Ram Karan, Anastassja Akal, Dominik Renn, Xuechao Liu, Seema Ghorpade, Michael Groll, Magnus Rueping, Jörg Eppinger

**Affiliations:** 1 King Abdullah University of Science and Technology (KAUST), KAUST Catalysis Center (KCC), Physical Sciences and Engineering Division (PSE), Thuwal, 23955-6900 Saudi Arabia; 2 Technical University of Munich (TUM), Center for Integrated Protein Science Munich in the Department Chemistry, Garching, Germany

**Keywords:** tRNAs, Protein design

## Abstract

The Pyrrolysyl-tRNA synthetase (PylRS) and its cognate tRNA^Pyl^ are extensively used to add non-canonical amino acids (ncAAs) to the genetic code of bacterial and eukaryotic cells. However, new ncAAs often require a cumbersome *de novo* engineering process to generate an appropriate PylRS/tRNA^Pyl^ pair. We here report a strategy to predict a PylRS variant with novel properties. The designed polyspecific PylRS variant HpRS catalyzes the aminoacylation of 31 structurally diverse ncAAs bearing clickable, fluorinated, fluorescent, and for the first time biotinylated entities. Moreover, we demonstrated a site-specific and copper-free conjugation strategy of a nanobody by the incorporation of biotin. The design of polyspecific PylRS variants offers an attractive alternative to existing screening approaches and provides insights into the complex PylRS-substrate interactions.

## Introduction

Site-specific incorporation of non-canonical amino acids (ncAAs) is a powerful tool to implement novel functions into the proteome^1–5^. The central element of a selective orthogonal ncAA incorporation system is an aminoacyl-tRNA synthetase/tRNA (aaRS/tRNA) pair that does not cross-react with the original aaRSs, tRNAs, or canonical amino acids of the host cell^6^. The pyrrolysyl-tRNA synthetase/tRNA^Pyl^ pair (PylRS/tRNA^Pyl^) has become a popular choice for the genetic code expansion^7,8^. This success is mainly attributed to the substrate flexibility of the active site^9,10^, the orthogonality in prokaryotic and eukaryotic cells^11^, and the inherent suppression of the amber codon^8^. Although over 150 ncAAs were genetically encoded during the past decade, these structures were mainly based on few core motifs^5^. Several structural functionalities still remain elusive, e.g., biotinylated ncAAs. The main factor limiting further expansion of the genetic code is the engineering of suitable aaRS/tRNA pairs. This process requires the construction of large mutant libraries from which all non-functional and non-orthogonal aaRS variants are eliminated through alternating rounds of positive and negative selection^2^. In most cases, selection screens are carried out as dead-and-alive assays on agar plates^2,12,13^. However, the selection conditions of this approach are hardly tunable, and therefore a beneficial aaRS might be accidentally eliminated^1,6,14^.

Screens based on fluorescence-activated cell sorting (FACS) provide a more sensitive and quantitative read-out and have been successfully applied to evolve the aaRS specificity^15–18^. However, the reported experimental setups involve several negative and positive selection rounds, resulting in a cumbersome process^17^.

Extensive efforts have been made to create a vast number of aaRS in the last years. These synthetases are often selected and used for the incorporation of a particular ncAA, although many engineered synthetases are polyspecific and aminoacylate a range of different ncAAs^19,20^. Hence, the aaRS-substrate information is not fully exploited and may be used to incorporate novel ncAAs, for which the de novo selection process had failed.

The current study investigated, whether the structural features of different PylRS variants can be recombined to a new synthetase exhibiting inherited and novel properties. Therefore, we created a mutant library based on reported PylRS variants and applied a sensitive selection screen. The identified PylRS-ncAA pairs provided insights for the prediction of the “highly polyspecific pyrrolysyl-tRNA synthetase” (HpRS). HpRS showed a tailored substrate scope and genetically incorporated for the first time biotin lysine. Furthermore, we demonstrate three different conjugation strategies of a GFP-nanobody using HpRS.

## Results and Discussion

### Library design

**Figure 1:**
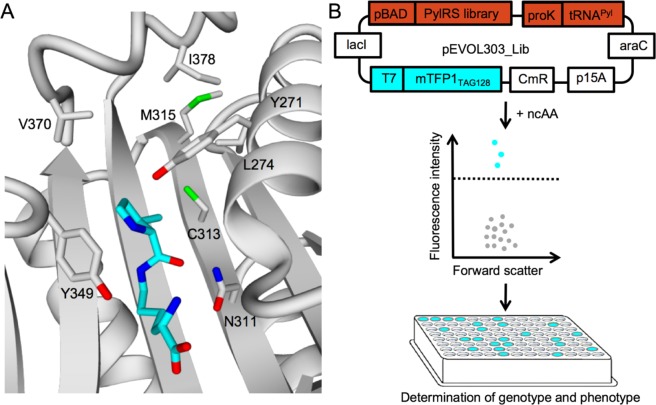
(**A**) Homology model of PylRS in complex with pyrrolysine (cyan) in the binding pocket. (**B**) Cells transformed with the plasmid pEVOL303_Lib are screened for ncAA incorporation by mTFP1-based fluorescence intensity and forward scatter using FACS. Fluorescent cells (cyan) exceeding a set threshold are sorted separately from nonfluorescent cells (grey) for sequencing on a 96-well plate. pEVOL303_Lib encodes for the PylRS library, tRNA^Pyl^ and, mTFP1_TAG128_. p15A origin of replication (p15A), *araC* repressor gene (araC), chloramphenicol acetyltransferase marker (CmR), *lac* repressor (lacI), T7 promoter (T7), proK promoter (proK), araBAD promoter (pBAD).

First, we designed a mutant library based on existing PylRS variants and a homology model of the *Methanosarcina barkeri* (*M. barkeri*) PylRS (Fig. 1A). The mutation Y349F enhanced the aminoacylation efficiency of PylRS and was included in the mutant library by default^10^. We maintained the orthogonality of the active site by retaining position N311 to eliminate a negative selection^21,22^. Based on these structural considerations, we selected the six amino acids Y271, L274, C313, M315, V370, and I378 to create a mutant PylRS library (Supplementary Table 1). In particular, smaller residues were inserted at these positions to increase the volume of the pocket and to allow the aminoacylation of larger ncAAs.

### Screening of PylRS variants

Dead-and-alive assays are the preferred method for screening large quantities of variants in a short time as demonstrated on existing aaRS/tRNA pairs^2,13^. Nevertheless, this screening method suffers from a harsh and hardly tunable selection pressure, causing slow cell growth and low survival rates^17^. Consequently, even beneficial variants might be unintentionally excluded from the library. Unlike dead-and-alive assays, fluorescence-activated cell sorting (FACS) provides a tunable sorting stringency over a broad dynamic range for the ncAA incorporation^15,17,23^.

**Figure 2:**
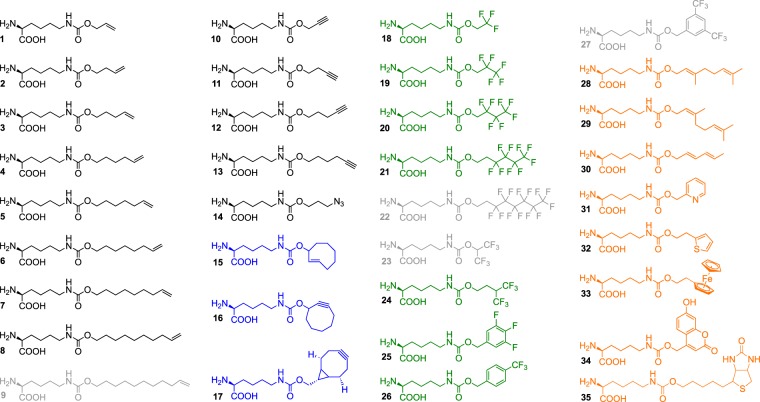
Chemical structures of analyzed ncAAs. Class I: linear alkenes, alkynes, and azide (black). Class II: cyclic alkenes and alkynes (blue). Class III: fluorinated side chains (green). Class IV: diverse functionalities (orange). Grey ncAA structures were rejected.

To set up a fast FACS screen, the PylRS library, tRNA^Pyl^ and the cyan monomeric teal fluorescent protein 1 (mTFP1) were expressed from a single plasmid (Fig. 1B). The mTFP1 contained an amber codon at position 128 (mTFP1_TAG128_) and allowed to quantify the incorporation efficiency of a ncAA according to the fluorescence intensity. *E. coli* cells transformed with pEVOL303_Lib were grown both in the absence and presence of ncAAs **1**–**23** (Fig. 2).

**Figure 3:**
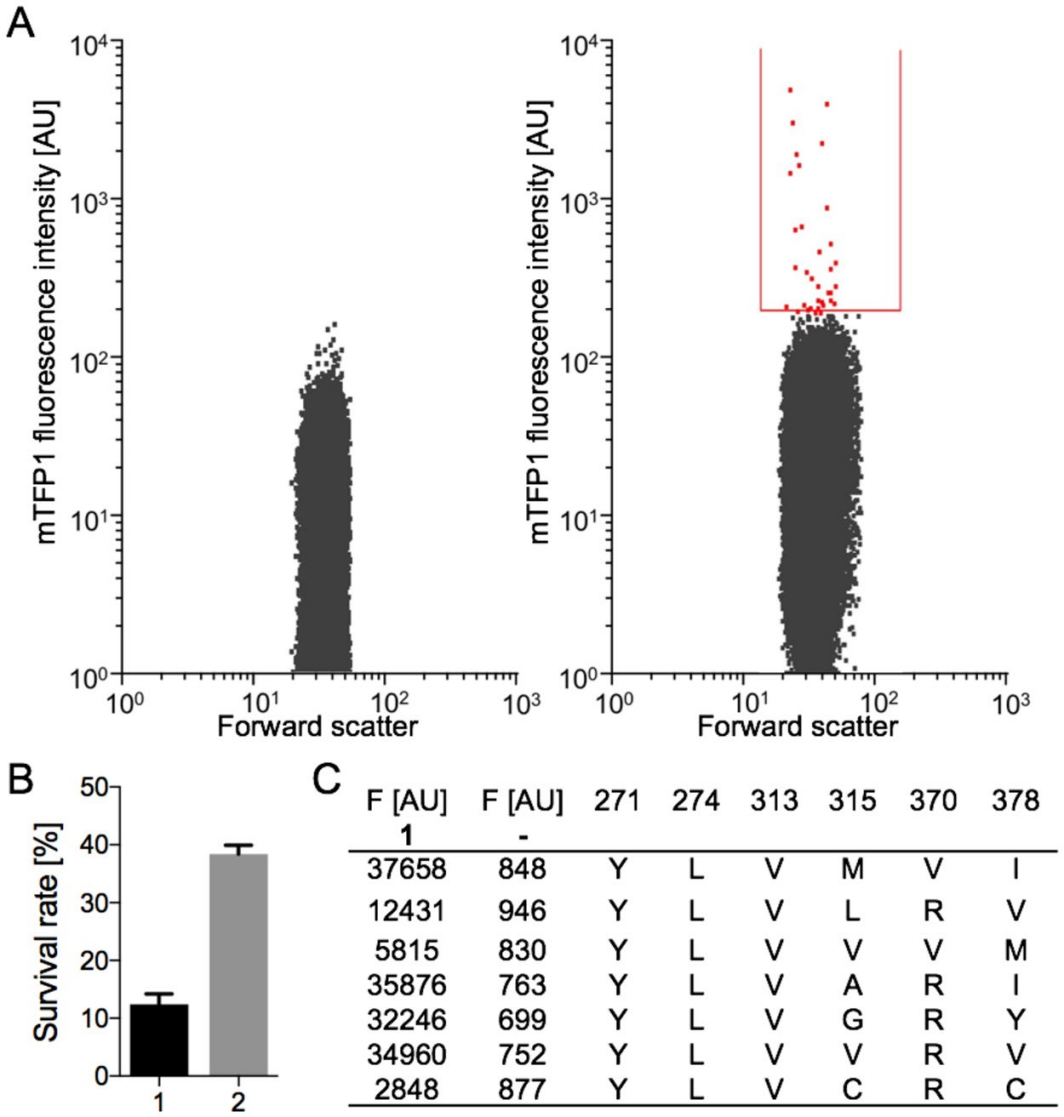
Identification of PylRS variants for the incorporation of ncAA **1**. (**A**) (Left plot) Fluorescence intensity and forward scatter of *E. coli* cells harboring pEVOL303_Lib in the absence of ncAAs analyzed by FACS. (Right plot) Cells harboring pEVOL303_Lib were grown in the presence of **1**. Fluorescent cells (red) within the sort gate (red line) were separated from nonfluorescent cells. (**B**) Survival rate of cells after FACS in LB medium (1) and conditioned medium (2). Results were obtained from five independent experiments; standard deviation is indicated by error bars. (**C**) Fluorescence intensities of mTFP1_TAG128_ expressed with **1** or without **–** in the present of seven different PylRS variants (mutations are shown).

Then, the forward scatter and fluorescent signals of each cell were analysed by FACS. In the presence of ncAA **1**, several cells depicted significant fluorescent signals (Fig. 3A, right plot) and negligible fluorescence in the absence of ncAAs indicates the orthogonality of the mutant library (Fig. 3A, left plot). Notably, the fluorescent cells appeared to be a heterogeneous population, suggesting that multiple PylRS variants enabled the aminoacylation of ncAA **1**. All fluorescent cells exceeding a set threshold were sorted on a 96-well plate supplied with conditioned medium, recovered overnight, and the PylRS variants were sequenced. The medium contained cell-secreted growth factors that improved the survival rate of single cells three-fold compared to the untreated lysogeny broth (LB) medium (Fig. 3B). In the case of ncAA **1**, seven different PylRS variants facilitated its incorporation (Fig. 3C). Next, expression of mTFP1_TAG128_ was repeated in the presence of the sequenced PylRS/tRNA^Pyl^ pairs and ncAA **1** to analyze the incorporation efficiency and orthogonality of the different variants (Fig. 3C).

**Figure 4:**
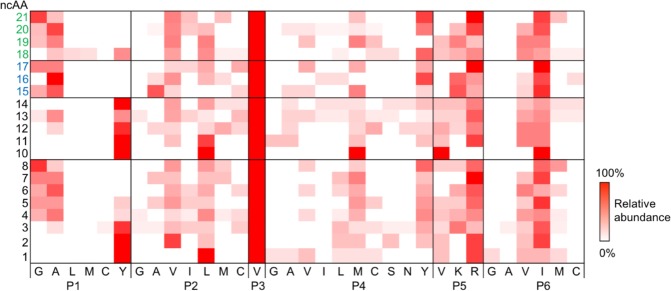
Heat map of the analyzed ncAAs **1–23** versus the six variable positions of the PylRS library (P1 = Y271, P2 = L274, P3 = C313, P4 = M315, P5 = V370, P6 = I378). Amino acids are represented by a one-letter code; the color intensity indicates the relative abundance of a mutation. Mutations not present in any of the sequenced PylRS variants have been omitted form the map. The ncAAs **9**, **22**, and **23** were not incorporated.

Overall, the screen identified 151 PylRS variants (110 unique and 41 redundant) that incorporated 20 out of the 23 initially tested ncAAs. The incorporation of ncAAs **9**, **22**, and **23** were not detected by FACS. Seventeen PylRS variants accepted multiple ncAAs. Individual ncAAs were aminoacylated by up to 16 different PylRS variants (Supplementary Table 3). The amino acid abundancies of the six mutated positions of all sorted PylRS variants are summarized for each ncAA in a heat map (Fig. 4). We divided the tested ncAAs **1**–**23** into three structural classes to elucidate key mutations in the binding pocket. Class I ncAAs **1**–**8** (alkenes) and **10**–**14** (alkynes) bear a linear aliphatic tail. Like a molecular ruler, these ncAAs allow to assess the maximum carbon chain length accepted by our PylRS library. Class II ncAAs **15**–**17** are cyclic and can be used in strain-promoted cycloadditions^24,25^. Highly fluorinated side chains of class III **18**–**23** might single out PylRS variants that accept bulky ncAAs, since the volume of a CF_3_ substituent (39.8 Å^3^) is about twice as big as a methyl-group (21.6 Å^3^)^26^.

### Pocket-substrate relationship

As expected, the 20 incorporated ncAAs required diverse structural adaptions of PylRS (Fig. 4). Y271 (P1) defines the bottom of the binding pocket; therefore, this position strongly varied with the ncAA size. While tyrosine was highly conserved at P1 for the short ncAAs **1**, **2**, **10**, **11**, and **14** alanine was favored by the larger ncAAs **4**–**7**, **13**, **15**–**17**, **19**, and **20**. Aminoacylation of the longest ncAAs **8** and **21** was preferred by glycine at P1 of the active site. We observed a similar trend for the position L274 (P2), which constitutes the rear end of the binding pocket. While leucine was favored by the small ncAAs **1**, **10**, and **11**, the shorter valine occurred in high abundance for larger ncAAs. Interestingly, the mutation C313V (P3), was strictly conserved throughout all sequenced variants. M315 (P4) differed widely for small ncAAs; however, tyrosine was more abundant among the larger ncAAs **8**, **16**, **20**, and **21**. V370 (P5) and I378 (P6) were not influenced by the size or chemistry of the incorporated ncAAs. Arginine and isoleucine respectively appeared with the highest frequency at these positions.

### Design of a polyspecific PylRS variant

**Figure 5:**
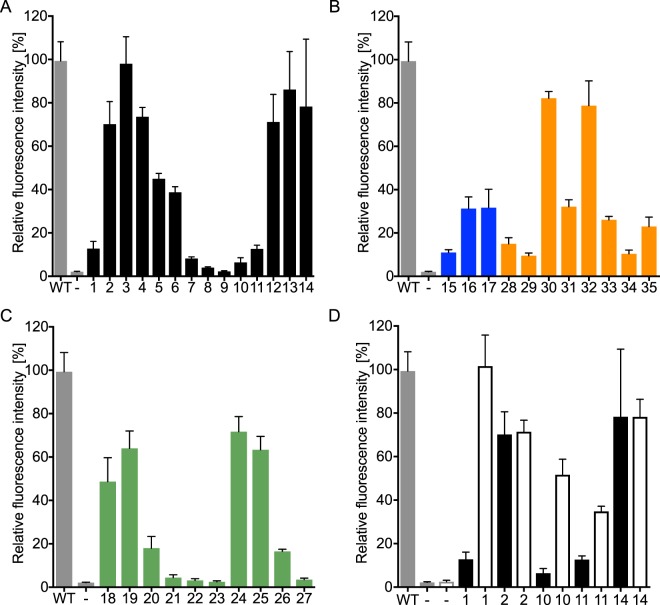
HpRS-dependent incorporation of ncAAs **1**–**35** in mTFP1_TAG128_. Fluorescence intensity of mTFP1 is normalized against wild-type (WT) mTFP1 (grey bar), while expression of mTFP1_TAG128_ in the absence of ncAAs (**−**, grey bar) served as a negative control. Error bars indicate the standard deviation of three independent experiments. (**A**) Substrate profiles of HpRS for class I (black): ncAAs **1**–**14**. (**B**) Class II (blue): ncAAs **15**–**17** and class IV (orange): ncAAs **28**–**35**. (**C**) Class III (green): ncAAs **18**–**27**. (**D**) Comparison of the aminoacylation efficiencies of HpRS (solid) and the PylRS variant (C313V, Y349F) (no fill) for ncAAs **1**, **2**, **10**, **11**, and **14**.

We combined potential key mutations Y271A, L274V, C313V, M315Y, Y349F, and V370R to design a highly polyspecific PylRS (HpRS) for the incorporation of large ncAAs. To test the substrate scope, the HpRS/tRNA^Pyl^ pair and mTFP1_TAG128_ were expressed with **1**–**35** in *E. coli*. The incorporation of the ncAAs **1**–**8**, **10**–**21**, **24**–**26**, **28**–**35** was detected by fluorescence signal (Fig. 5) and additionally confirmed by SDS-PAGE (Supplementary Fig. 1) and ESI-TOF (Supplementary Table 4, Supplementary Fig. 2). Further, we used Alexa Fluor 532 streptavidin conjugate to confirm the incorporation of ncAA **35** in mTFP1_TAG128_ (Supplementary Fig. 3).

**Figure 6:**
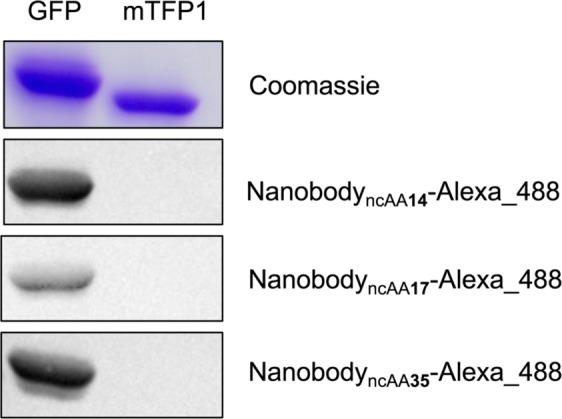
Coomassie stained SDS gel of cell lysates containing GFP or mTFP1 and western blot on the same cell lysates with an anti-GFP nanobody bearing ncAA **14**, **17** or **35** conjugated to Alexa Fluor 488.

Intriguingly, HpRS tolerated a broad range of lysine carbamates with diverse features, including aliphatic chains, fluorinated residues, cyclic structures, and a biotinylated ncAA. As predicted, HpRS was highly capable of aminoacylation of ncAAs with long side chains **3-6**; yet, the smallest tested ncAAs were only moderately accepted (Fig. 5). Therefore, the mutations C313V and Y349F were combined based on the heat map to generate a PylRS particular suited for small ncAAs. Indeed, this variant showed a significant higher aminoacylation efficiency of the small ncAAs **1**, **10**, and **11** than HpRS (Fig. 6D).

The polyspecific PylRS containing the mutations Y271A and Y349F has been reported for **15**–**17**^24,27^. To compare HpRS with PylRS (Y271A, Y349F), both synthetases were co-expressed with mTFP1_TAG128_ in the presence of **15**–**17** and the fluorescence signals were analysed. The ncAAs were incorporated in mTFP1_TAG128_ in equivalent yields by both synthetases (Supplementary Fig. 4).

### Labeling of nanobodies

Immunoassays such as flow cytometry or western blot usually require commercially available antibodies-conjugates. In contrast, nanobodies derived from Camelid antibodies, offer nowadays an attractive alternative^28^, as they are highly stable, small and can be expressed in heterologous systems such as *E. coli* in high yields^29^. The developed HpRS/tRNA^Pyl^ pair was employed to incorporate ncAA **14**, **17**, **35** in an anti-GFP nanobody, which exhibits a specific binding activity against the green fluorescence protein (GFP)^30^, at the exposed position K44 with expression yields of 8 mg/L, 4 mg/L, 4 mg/L in *E. coli* culture, respectively. The ncAA incorporation into anti-GFP nanobody_TAG44_ was confirmed by SDS-PAGE (Supplementary Fig. 5) and LC-MS analysis. To conjugate the anti-GFP nanobody_ncAA14_, the applied azide was linked to an Alexa Fluor 488 alkyne via copper-catalyzed azide-alkyne “click” reaction. The bicyclononyne-lysine (BCN), ncAA **17**, in the anti-GFP nanobody_ncAA17_ formed  a strain-promoted [3 + 2] azide-alkyne cycloaddition with an Alexa Fluor 488 azide. The biotin containing anti-GFP nanobody_ncAA35_ bound successfully an Alexa Fluor 488 marked streptavidin.

A western blot of cell lysates containing GFP or mTFP1, as a negative control, demonstrated the specific detection of GFP by the established anti-GFP nanobody-conjugates (Fig. 6). Notably, the most efficient coupling reaction between anti-GFP nanobody and fluorescence-dye seemed to be the copper-catalyzed “click” reaction and the biotin-streptavidin interaction, thus resulting in the strongest western blot signal (Fig. 6).

In summary, we demonstrated that the PylRS-substrate interaction can be used to recombine key mutation to generate new PylRS variants. This seems at first glance a limiting engineering approach, however as a proof of concept the designed HpRS exhibit a remarkable substrate scope and facilitates the incorporation of a biotin bearing ncAA. A prerequisite for this method is the generation of a diverse pool of beneficial PylRS variants that was achieved by a fast and mild FACS setup. PylRS diversity for a particular ncAA provided insights into the complex PylRS-substrate interaction and allowed the prediction of polyspecific variants and can now be used as an example for others to avoid the cumbersome engineering process.

Moreover, HpRS was used to insert **14**, **17** or **35** in an anti-GFP nanobody. The three different chemical handles were exploited to produce fluorescent nanobody-conjugates. In particular, the streptavidin-biotin interaction allows efficient labeling of the anti-GFP nanobody without a catalyst. Therefore, the incorporation of **35** might be a good starting point to simplify techniques such as western blotting, ELISA, flow cytometry or antibody labeling in general.

## Methods

### Strains

All cloning steps were performed in TOP10 *E. coli* (Thermo Fisher Scientific). The FACS and protein expression experiments were carried out with BL21 (DE3) (Lucigen, Middleton, WI).

### Medium

A conditioned medium was used to improve the survival of sorted cells after FACS. To prepare the conditioned medium, BL21 (DE3) cultures were grown in LB (OD_600_~1.0), centrifuged at 8000 × g for 20 min at 4 °C and the supernatant was sterilized by passing through a 0.22 μm cellulose filter (Millipore, Bedford, MA).

### Non-canonical amino acids (ncAA)

ncAA **1** (Sigma-Aldrich), ncAA **15**–**17** (Sirius Fine Chemicals, Bremen, Germany), and ncAAs **24**–**27**, **30**–**33**, as well as **35** (SUNGYOUNG Chemical Limited, Shanghai, China) were purchased from commercial sources. ncAA **34** was synthesized according to the procedure published by Luo *et al*.^31^ ncAA **2**–**14**, **18**–**23**, **28**, and **29** were synthesized according to a modified procedure from Li *et al*.^32^ (Supplementary Information, S2).

### Plasmid construction

The plasmid pEVOL303 was constructed by ligating the plasmid pEVOL^33^ with the commercially available pET303/CT-His (Thermo Fisher Scientific) using Gibson cloning^34^. Prior to ligation, pEVOL was digested with the restriction enzymes *Sac*I and *Pci*I. The required region of pET303 was amplified using the primers pET303_f and pET303_r (Supplementary Table 2). The resulting plasmid, pEVOL303, carried a p15A origin, a chloramphenicol resistance gene, a *Methanosarcina barkeri* PylRS gene downstream of a pBAD promoter, and the corresponding *tRNA* gene controlled by a proK promoter. The fluorescent protein gene with an N-terminal 6xHis-SUMO-tag, either wild-type mTFP1 or mTFP1_TAG128_ (bearing an amber codon at position 128), was cloned downstream of a T7 promoter. The amber codon was inserted using the primers mTFP_128_f and mTFP_128_r (Supplementary Table 2). The GFP-nanobody was encoded on the plasmid pOPINE^30^. The amber codon was inserted at position 44 using the primers GFP_Nano_f and GFP_Nano_r (Supplementary Table 2).

### PylRS library construction

#### Methanosarcina barkeri

PylRS containing the single mutation Y349F served as the starting point for our mutant library. Six positions in PylRS, Y271, L274, C313, M315, V370, and I378, were selected for our library based on a homology model derived from the crystal structure of PylRS (PDB 4Q6G and 4CS3) (Fig. 1A,B)^35,36^. A pre-defined set of amino acids was inserted into the six positions, resulting in 95,040 mutants. To ensure an equal distribution of all the mutants, the library was assembled non-codon redundant and codon usage optimized by the company, Life Technologies (Thermo Fisher Scientific) and introduced to pEVOL303 *via* the restriction sites *Sal*I and *Bgl*II. The resulting plasmid, pEVOL303_Lib, was transformed into BL21 (DE3) cells by applying the standard electroporation protocol. The transformation was determined with 1 × 10^7^ CFU per preparation.

### Sample preparation for FACS

BL21 (DE3) cells transformed with pEVOL303_Lib were inoculated in 50 mL LB medium supplemented with 25 µg/mL chloramphenicol (Cm), and grown until an OD_600_ of 0.7. Then, 450 µL of the cells was transferred into a 2 mL reaction vessel and induced with 50 µL induction medium (10 mM ncAA, 10 mM IPTG, 1% w/v arabinose) and incubated for 16 h at 37 °C and 700 rpm. The cells were diluted to a final concentration of 1 × 10^7^ cell/mL and washed twice with an M9 minimal medium supplemented with 25 µg/mL chloramphenicol. In the beginning of each FACS experiment, the negative control (cell growth in the absence of ncAA) was screened first to ensure the discrimination of canonical amino acids by the PylRS mutant library. Cell sorting was performed by detecting the fluorescence of mTFP1 with a BD Influx (BD Biosciences) operated with filter-sterilized BD FACS Flow Sheath Fluid, a 457 nm laser for excitation, and a 480/40 bandpass filter. The selected operation mode was 1.0 Drop Single. The selection threshold (gate) was adjusted based on the fluorescent signal from the first 1 × 10^5^ cells of each sample. Cells within the gate were sorted separately on a 96-well plate (Sigma-Aldrich) supplied with the conditioned medium containing 25 µg/mL chloramphenicol. The growth of the sorted cells was continued overnight at 37 °C and 300 rpm. Each culture was sequenced using the primers Lib_seq_f and Lib_seq_r (Supplementary Table 2). pEVOL303_Lib containing the sequenced PylRS variants were retransformed into new BL21 (DE3) cells, grown and induced with the induction medium at OD_600_ of 0.7. Correspondingly, PylRS, tRNA^Pyl^, and mTFP1_TAG128_ were expressed. Both the incorporation of the ncAAs and the orthogonality of the PylRS variant were confirmed by fluorescence measurement of mTFP1 (excitation 462 nm, emission 492 nm) in a black, 96-well plate (Thermo Fisher Scientific) with an Infinite M1000 plate reader (Tecan, Zurich, Switzerland).

### ncAA incorporation

A single BL21 (DE3) colony containing pEVOL303 encoding for HpRS, PylRS (Y271A and Y349F) or PylRS (C313V and Y349F) was selected from the LB/Cm agar plate and inoculated into 50 mL LB/Cm media, then incubated and shaken overnight at 37 °C. The resulting culture was diluted (1:100) with fresh LB/Cm media and grown to an OD_600_ of 1.5. The mTFP1 expression was triggered by adding 1 mM ncAA, 1 mM IPTG, and 0.1% w/v arabinose and cell growth continued at 37 °C and 700 rpm for 16 h. Fluorescence was measured correspondingly. For SDS-PAGE, the sample was heated at 75 °C for 20 min and cleared by centrifugation. For protein purification, cell pellet was suspended in lysis buffer (100 mM Tris pH 7.5, 500 mM NaCl, 20 mM imidazole, 10%, v/v glycerol), incubated at 75 °C for 20 min and cleared by centrifugation

The supernatant was loaded onto a HisTrap HP Ni–NTA column (GE Healthcare). Protein eluted by increasing the concentration of imidazole from 20 mM to 500 mM in the lysis buffer over 10 column volumes. SUMO protease was added to the eluted protein, dialyzed overnight at 4 °C against ddH_2_O and passed through a HisTrap HP Ni–NTA to remove the remaining N-terminal 6xHis- as well as the SUMO-tag.

The HpRS/tRNA^Pyl^ pair was employed to incorporate the respective ncAAs at position K44 of the reported anti-GFP nanobody^30^. BL21(DE3) were grown at 37 °C to OD_600_ 0.7 in 500 mL LB medium. Protein synthesis was induced by 0.5 mM IPTG, 0.1% w/v arabinose, and 1 mM ncAA. Propagation was carried out at 20 °C for 20 h. Cells were harvested by centrifugation, lysed by sonication and cleared by centrifugation. The cell lysate was applied onto a HisTrap HP Ni–NTA column and eluted in 50 mM Tris-HCl pH 7.5, 300 mM NaCl, 1 mM TCEP, 500 mM Imidazole. The eluted protein was concentrated and loaded onto a HiLoad Superdex 200 (16/60) size-exclusion column (GE Healthcare) equilibrated with 50 mM Tris-HCl pH 7.5, 150 mM NaCl, 1 mM TCEP.

### Conjugation of nanobodies

500 nM purified GFP-nanobody containing **14**, **17** or **35** was labeled with either Alexa 488 alkyne (40 µM, 1 h) using the Click-iT Protein Reaction Buffer Kit, Alexa 488 azide (40 µM, 6 h) or Alexa 488 streptavidin (5 µM, 1 h) at 37 °C (Thermo Fisher Scientific).

### Western blot

*E. coli* cell lysate containing GFP or mTFP1 was electrophoretically fractionated and electroblotted onto 0.45 μm Immobilon-P polyvinylidene difluoride (PVDF) membranes (Merck, Darmstadt, Germany). After electroblotting, the membranes were washed three times for 5 min with TBS buffer and blocked in TBST (10 mM Tris, 0.9%, w/v NaCl, 0.05%, v/v, Tween-20, pH 7.4) with 1% (w/v) casein for 3 h at room temperature. The blots were probed and incubated with the conjugated GFP-nanobodies (1 µg/mL) at room temperature for 60 min. The washing steps were performed with TBST. Fluorescence was detected in an iBright FL1000 imaging system (Invitrogen).

### Mass spectrometry

Pure mTFP1 and GFP-nanobodies were analysed by mass spectrometry (maXis HD ESI-TOF, Bruker). The sample was injected into a high-performance liquid chromatography (Agilent Technologies, C4 column, column volume of 5 mL) and separation was performed at a constant flow rate of 0.5 µL/min and a gradient of 80% acetonitrile and 0.1% formic acid for 8 min. Fractions were recorded according to the standard procedure.

### Homology model of the *Methanosarcina barkeri* PylRS in complex with pyrrolysine

Homology models were generated using the YASARA Structure, Version 14.7.17^37^. The catalytic domain of *Methanosarcina barkeri* PylRS served as the template for YASARA’s homology modeling macro, using the conservative “slow” protocol with the following parameter settings: number of PSI-BLAST iterations – 10; maximum allowed BLAST E-value to consider template – 0.5; maximum oligomerization state – 2; maximum number of alignment variations per template – 4; maximum number of conformations tried per loop – 200; and maximum number of residues added to the termini – 20. The resulting homology model was based on two structures of PylRS variants, PDB 4Q6G and 4CS3; both sequences had a 98% homology with the template^35,36^. The YASARA algorithm performed the secondary structure prediction, loop construction, and amino-acid rotamer selection, followed by a steepest-descent energy minimization.

## Supplementary information

Engineering a Polyspecific Pyrrolysyl-tRNA Synthetase by a High Throughput FACS Screen
