# Supplementary material for: Engineering a Polyspecific Pyrrolysyl-tRNA Synthetase by a High Throughput FACS Screen
Source: Sci Rep. 2019 Aug 19;9:11971. doi: 10.1038/s41598-019-48357-0 (PMC6700097; doi:10.1038/s41598-019-48357-0)
Supplement: Supplementary file 1 — Engineering a Polyspecific Pyrrolysyl-tRNA Synthetase by a High Throughput FACS Screen [file 41598_2019_48357_MOESM1_ESM.docx]

**SUPPLEMENTARY INFORMATION**

**Engineering a Polyspecific Pyrrolysyl-tRNA Synthetase by a High Throughput FACS Screen**

**Adrian Hohl**^∞^**^1,2^, Ram Karan**^∞^**^1*^, Anastassja Akal^1,2^, Dominik Renn^1,2^, Xuechao Liu^1^, Seema Ghorpade^1^, Michael Groll^2^, Magnus Rueping^1*^, Jörg Eppinger^1*^**

∞contributed equally

**^1^** King Abdullah University of Science and Technology (KAUST), KAUST Catalysis Center (KCC), Physical Sciences and Engineering Division (PSE), Thuwal, 23955-6900, Saudi Arabia

^2^Technical University of Munich, Center for Integrated Protein Science Munich in the Department Chemistry, Garching, Germany

**^*^**For correspondence: ram.karan@kaust.edu.sa, magnus.rueping@kaust.edu.sa, [jorg.eppinger@kaust.edu.sa](mailto:jorg.eppinger@kaust.edu.sa)

**S1. Additional Tables and Figures**

Supplementary Table 1. Mutation scheme of the synthesized mutant library. The mutation Y349F was included in the mutant library by default.

**
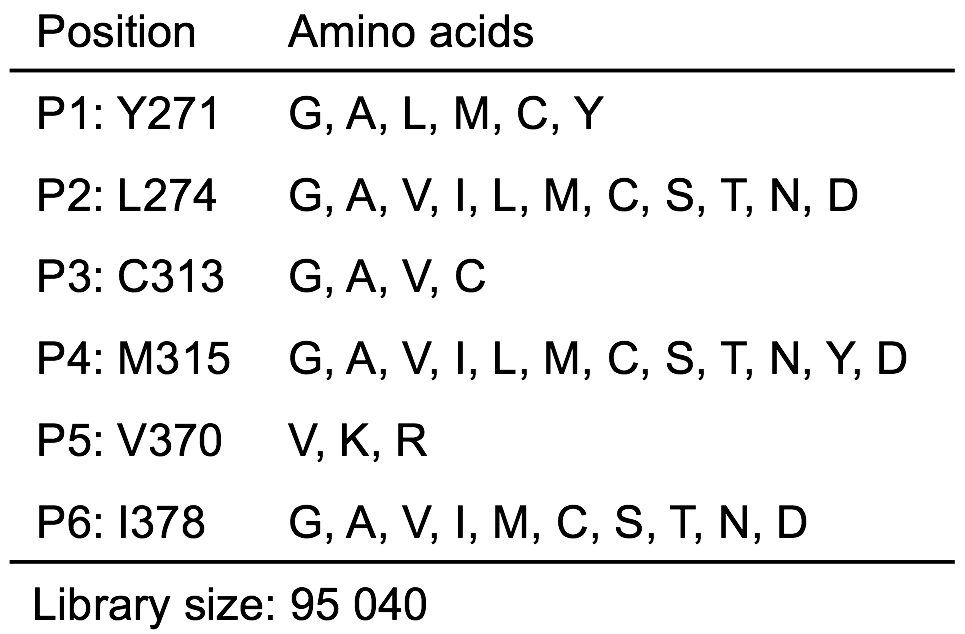
**

**Supplementary Table 2.** List of oligomers used in this study.

| Description | Sequence (5’-3’) |
| --- | --- |
| pET303_f | GAAAGGCTCAGTCGAAAGACTGGGCCTTGTTTGTGAGCTTAGTCATGCCCCGCGCC |
| pET303_r | CGGTCACACTGCTTCCGGTAGTCAATAAACCGGTGCTCATCAGCAAAAAACCCCTCAAGACC |
| Lib_seq_f | AGATCTATGGACAAAAAACCGCTG |
| Lib_seq_r | GTCGACTTACAGGTTGGTAG |
| mTFP_128_f | CGAGAACTTCCCCCCCTAGGGCCCCGTG |
| mTFP_128_r | CACGGGGCCCTAGGGGGGGAAGTTCTCG |
| GFP_Nano_f | TCGCCAGGCGCCTGGTTAGGAACGTGAATGGGTTG |
| GFP_Nano_r | CAACCCATTCACGTTCCTAACCAGGCGCCTGGCGA |

**Supplementary Table 3.** List of 110 unique PylRS variants and their corresponding substrate scopes with respect to the ncAAs. A total of 151 PylRS variants were sequenced. Only the six positions in PylRS that were included in the mutation library are depicted. The normalized fluorescence intensity of mTFP1_TAG128_ (F [%]) co-expressed with a PylRS/tRNA^Pyl^ pair, is listed both with (+) and without (-) ncAAs (1 mM). Fluorescence intensity was normalized against wild-type mTFP1. PylRS variants were not detected for ncAAs **9**, **22**, or **23**.

|  | Position | | | | |  |  |  |  |
| --- | --- | --- | --- | --- | --- | --- | --- | --- | --- |
|  | P1 | P2 | P3 | P4 | P5 | P6 |  |  |  |
| Variant | 271 | 274 | 313 | 315 | 370 | 378 | ncAA | F [%] + | F [%] - |
| 1 | Y | L | V | M | V | I | 1 | 94 | 2 |
|  |  |  |  |  |  |  | 4 | 33 | 2 |
|  |  |  |  |  |  |  | 10 | 48 | 1 |
|  |  |  |  |  |  |  | 12 | 35 | 2 |
|  |  |  |  |  |  |  | 13 | 23 | 2 |
|  |  |  |  |  |  |  | 14 | 87 | 1 |
| 2 | Y | L | V | L | R | V | 1 | 31 | 2 |
| 3 | Y | L | V | V | V | M | 1 | 15 | 2 |
| 4 | Y | L | V | A | R | I | 1 | 90 | 2 |
| 5 | Y | L | V | G | R | Y | 1 | 81 | 2 |
| 6 | Y | L | V | V | R | V | 1 | 87 | 2 |
| 7 | Y | L | V | C | R | C | 1 | 7 | 2 |
| 8 | Y | V | V | S | R | I | 2 | 32 | 2 |
| 9 | Y | V | V | L | R | V | 2 | 27 | 1 |
| 10 | Y | V | V | M | V | I | 2 | 68 | 1 |
| 11 | Y | V | V | Y | R | I | 2 | 32 | 1 |
|  |  |  |  |  |  |  | 11 | 90 | 2 |
| 12 | Y | A | V | N | K | A | 3 | 3 | 2 |
| 13 | C | L | V | L | R | I | 3 | 11 | 2 |
| 14 | Y | V | V | S | K | I | 3 | 45 | 2 |
| 15 | Y | I | V | L | K | I | 3 | 42 | 2 |
| 16 | Y | C | V | C | K | V | 3 | 75 | 2 |
| 17 | A | L | V | Y | K | M | 3 | 22 | 2 |
|  |  |  |  |  |  |  | 18 | 45 | 2 |
| 18 | Y | C | V | L | K | I | 3 | 75 | 2 |
| 19 | Y | V | V | Y | K | I | 3 | 68 | 2 |
| 20 | Y | G | V | Y | R | V | 3 | 27 | 2 |
| 21 | Y | V | V | M | R | I | 3 | 71 | 2 |
| 22 | A | C | V | L | V | I | 3 | 26 | 2 |
| 23 | Y | I | V | Y | K | I | 3 | 70 | 2 |
| 24 | Y | I | V | Y | V | I | 3 | 78 | 2 |
| 25 | Y | V | V | C | R | V | 3 | 74 | 2 |
|  |  |  |  |  |  |  | 18 | 38 | 2 |
| 26 | A | V | V | Y | R | I | 3 | 108 | 2 |
|  |  |  |  |  |  |  | 5 | 44 | 2 |
|  |  |  |  |  |  |  | 7 | 13 | 2 |
|  |  |  |  |  |  |  | 16 | 33 | 2 |
|  |  |  |  |  |  |  | 18 | 57 | 2 |
|  |  |  |  |  |  |  | 20 | 21 | 2 |
|  |  |  |  |  |  |  | 20 | 17 | 2 |
|  |  |  |  |  |  |  | 21 | 5 | 2 |
| 27 | A | I | V | V | R | V | 4 | 56 | 2 |
|  |  |  |  |  |  |  | 13 | 5 | 1 |
|  |  |  |  |  |  |  | 20 | 7 | 2 |
| 28 | Y | C | V | M | V | V | 4 | 90 | 2 |
| 29 | G | L | V | M | K | V | 4 | 61 | 2 |
| 30 | A | L | V | Y | K | V | 4 | 71 | 2 |
|  |  |  |  |  |  |  | 16 | 22 | 2 |
|  |  |  |  |  |  |  | 20 | 11 | 2 |
| 31 | A | L | V | Y | R | I | 4 | 77 | 3 |
| 32 | G | L | V | Y | R | I | 4 | 54 | 2 |
|  |  |  |  |  |  |  | 17 | 22 | 2 |
| 33 | Y | G | V | V | R | I | 4 | 79 | 2 |
| 34 | A | V | V | L | R | I | 4 | 70 | 2 |
|  |  |  |  |  |  |  | 6 | 6 | 2 |
|  |  |  |  |  |  |  | 7 | 6 | 2 |
|  |  |  |  |  |  |  | 8 | 5 | 2 |
|  |  |  |  |  |  |  | 20 | 6 | 2 |
| 35 | G | M | V | M | V | I | 4 | 45 | 2 |
| 36 | A | V | V | M | R | I | 4 | 78 | 2 |
| 37 | A | C | V | Y | K | M | 4 | 68 | 2 |
|  |  |  |  |  |  |  | 13 | 64 | 2 |
| 38 | A | L | V | Y | V | V | 4 | 67 | 2 |
| 39 | G | L | V | M | R | I | 5 | 49 | 2 |
|  |  |  |  |  |  |  | 7 | 8 | 2 |
|  |  |  |  |  |  |  | 8 | 4 | 2 |
|  |  |  |  |  |  |  | 17 | 32 | 2 |
|  |  |  |  |  |  |  | 17 | 33 | 2 |
| 40 | Y | A | V | C | V | V | 5 | 28 | 2 |
| 41 | G | V | V | M | R | I | 5 | 36 | 2 |
| 42 | A | I | V | Y | K | I | 5 | 25 | 2 |
|  |  |  |  |  |  |  | 20 | 10 | 2 |
| 43 | G | L | V | V | R | V | 6 | 29 | 2 |
| 44 | A | L | V | M | K | M | 6 | 17 | 2 |
| 45 | A | C | V | Y | R | V | 6 | 33 | 2 |
| 46 | A | I | V | M | K | V | 6 | 21 | 2 |
| 47 | G | V | V | Y | R | I | 6 | 37 | 2 |
|  |  |  |  |  |  |  | 8 | 4 | 2 |
|  |  |  |  |  |  |  | 20 | 19 | 2 |
|  |  |  |  |  |  |  | 20 | 24 | 2 |
|  |  |  |  |  |  |  | 20 | 14 | 2 |
|  |  |  |  |  |  |  | 21 | 3 | 2 |
|  |  |  |  |  |  |  | 21 | 3 | 2 |
| 48 | G | M | V | M | R | I | 7 | 5 | 2 |
|  |  |  |  |  |  |  | 8 | 4 | 2 |
|  |  |  |  |  |  |  | 21 | 5 | 2 |
| 49 | A | A | V | Y | R | V | 7 | 5 | 2 |
|  |  |  |  |  |  |  | 8 | 9 | 2 |
| 50 | G | M | V | M | R | V | 7 | 5 | 2 |
| 51 | Y | L | V | M | R | I | 11 | 38 | 2 |
| 52 | Y | L | V | A | R | V | 11 | 39 | 2 |
| 53 | Y | L | V | G | R | V | 11 | 13 | 2 |
| 54 | Y | C | V | C | V | V | 12 | 40 | 2 |
| 55 | Y | M | V | C | K | V | 12 | 11 | 2 |
| 56 | Y | V | V | N | R | I | 12 | 10 | 2 |
| 57 | A | M | V | Y | R | V | 12 | 23 | 2 |
| 58 | Y | A | V | A | R | I | 12 | 24 | 2 |
| 59 | A | I | V | M | R | V | 13 | 32 | 2 |
| 60 | Y | Y | V | V | R | V | 13 | 23 | 2 |
| 61 | Y | V | V | A | R | C | 13 | 30 | 2 |
| 62 | Y | C | V | S | R | I | 13 | 40 | 2 |
| 63 | G | V | V | L | V | I | 13 | 16 | 2 |
| 64 | Y | G | V | A | R | V | 13 | 69 | 2 |
| 65 | A | C | V | I | R | I | 13 | 53 | 2 |
| 66 | A | V | V | C | K | I | 13 | 45 | 2 |
|  |  |  |  |  |  |  | 20 | 6 | 2 |
| 67 | Y | V | V | L | R | I | 14 | 87 | 1 |
| 68 | Y | L | V | I | R | I | 14 | 81 | 2 |
| 69 | Y | M | V | N | R | V | 14 | 57 | 2 |
| 70 | Y | V | V | A | V | M | 14 | 74 | 2 |
| 71 | Y | V | V | Y | K | M | 14 | 73 | 2 |
| 72 | Y | C | V | S | R | C | 14 | 31 | 2 |
| 73 | Y | L | V | Y | K | V | 14 | 26 | 1 |
| 74 | G | C | V | Y | K | I | 15 | 7 | 2 |
| 75 | A | A | V | V | K | I | 15 | 7 | 2 |
| 76 | A | A | V | L | K | I | 15 | 6 | 2 |
| 77 | G | A | V | L | R | I | 15 | 8 | 2 |
| 78 | A | A | V | L | R | V | 15 | 8 | 2 |
| 79 | A | V | V | A | K | I | 15 | 8 | 2 |
| 80 | A | A | V | I | K | I | 16 | 10 | 2 |
| 81 | A | L | V | Y | K | I | 16 | 16 | 2 |
|  |  |  |  |  |  |  | 18 | 29 | 2 |
| 82 | A | V | V | L | K | I | 16 | 13 | 2 |
| 83 | A | V | V | Y | R | C | 16 | 25 | 2 |
| 84 | A | M | V | Y | R | I | 16 | 11 | 2 |
| 85 | A | C | V | Y | R | I | 17 | 34 | 2 |
| 86 | A | C | V | V | R | I | 17 | 28 | 2 |
| 87 | G | I | V | C | R | I | 17 | 27 | 2 |
| 88 | M | L | V | Y | R | I | 18 | 36 | 2 |
| 89 | Y | C | V | Y | R | C | 18 | 32 | 2 |
| 90 | L | M | V | Y | R | I | 18 | 29 | 2 |
| 91 | Y | V | V | V | R | V | 18 | 37 | 2 |
| 92 | F | V | V | S | V | V | 18 | 4 | 2 |
| 93 | Y | L | V | L | K | V | 18 | 65 | 2 |
| 94 | Y | V | V | Y | K | V | 18 | 25 | 2 |
| 95 | M | L | V | Y | V | I | 18 | 30 | 2 |
| 96 | Y | L | V | S | R | V | 18 | 47 | 2 |
| 97 | A | L | V | V | K | V | 19 | 45 | 2 |
| 98 | Y | V | V | C | R | I | 19 | 25 | 2 |
| 99 | G | L | V | M | V | I | 19 | 53 | 2 |
| 100 | A | L | V | M | K | V | 19 | 63 | 2 |
| 101 | A | L | V | A | R | I | 20 | 6 | 2 |
| 102 | A | I | V | A | R | I | 20 | 7 | 2 |
| 103 | A | L | V | Y | R | V | 20 | 24 | 2 |
| 104 | G | L | V | M | K | I | 20 | 12 | 2 |
| 105 | G | I | V | Y | R | V | 20 | 5 | 2 |
| 106 | A | L | V | M | R | V | 20 | 11 | 2 |
| 107 | A | V | V | V | K | I | 20 | 8 | 2 |
| 108 | A | A | V | I | R | I | 20 | 5 | 2 |
| 109 | A | V | V | Y | R | V | 20 | 12 | 2 |
| 110 | A | I | V | Y | R | M | 21 | 4 | 2 |

**Supplementary Table 4.** ESI-TOF data from purified wild-type mTFP1 (WT) and mTFP1 containing ncAAs **1–35**. For exemplary ESI-MS spectra see Supplementary Figure 2.

| ncAA | Theoretical Mass (Da) | Experimental Mass (Da) | ± Mass (Da) |
| --- | --- | --- | --- |
| WT^a^ | 25154.5 | 25154.7 | 0.2 |
| 1 | 25252.6 | 25251.8 | 0.8 |
| 2 | 25266.6 | 25265.1 | 1.5 |
| 3 | 25280.7 | 25280.2 | 0.5 |
| 4 | 25294.7 | 25294.4 | 0.3 |
| 5 | 25308.7 | 25306.2 | 2.5 |
| 6 | 25322.7 | 25320.9 | 1.8 |
| 7 | 25336.8 | 25334.9 | 1.9 |
| 8 | 25350.8 | 25349.3 | 1.5 |
| 9 | 25364.8 | NA | NA |
| 10 | 25250.6 | 25250.2 | 0.4 |
| 11 | 25264.6 | 25264.4 | 0.2 |
| 12 | 25278.6 | 25277.3 | 1.3 |
| 13 | 25292.7 | 25291.4 | 1.3 |
| 14 | 25295.6 | 25293.4 | 2.2 |
| 15 | 25320.7 | 25318.9 | 1.8 |
| 16 | 25318.7 | 25318.5 | 0.2 |
| 17 | 25344.7 | 25342.3 | 2.4 |
| 18 | 25294.6 | 25293.1 | 1.5 |
| 19 | 25344.6 | 25342.2 | 2.4 |
| 20 | 25394.6 | 25394.6 | 0.0 |
| 21 | 25458.6 | 25457.1 | 1.5 |
| 22 | 25558.6 | NA | NA |
| 23 | 25362.6 | NA | NA |
| 24 | 25390.6 | 25389.1 | 1.5 |
| 25 | 25356.6 | 25355.5 | 1.1 |
| 26 | 25370.7 | 25368.5 | 2.2 |
| 27 | 25438.7 | NA | NA |
| 28 | 25348.8 | 25347.8 | 1.0 |
| 29 | 25348.8 | 25347.5 | 1.3 |
| 30 | 25292.7 | 25290.5 | 2.2 |
| 31 | 25303.6 | 25302.1 | 1.5 |
| 32 | 25322.7 | 25321.4 | 1.3 |
| 33 | 25438.6 | 25437.1 | 1.5 |
| 34 | 25386.7 | 25384.6 | 2.1 |
| 35 | 25424.8 | 25423.9 | 0.9 |

1. WT has the amino acid asparagine (Asn) in position 128.


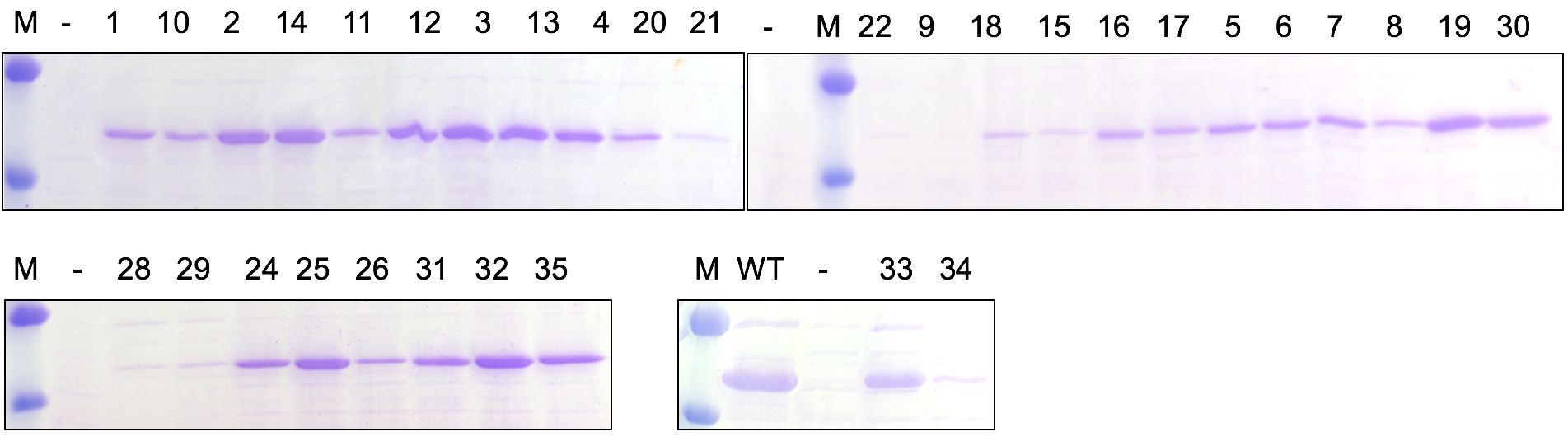


**Supplementary Figure 1.** SDS-PAGE analysis of the heat-treated *E. coli* lysate containing mTFP1 with an N-terminal His_6_-SUMO tag. mTFP1_TAG128_ was co-expressed with the HpRS/tRNA^Pyl^ pair, both with and without ncAAs **1**–**35** (1 mM). ncAA **23** and **27** were not incorporated into the mTFP1 (data not shown). Marker bands indicate 36 and 55 kDa.


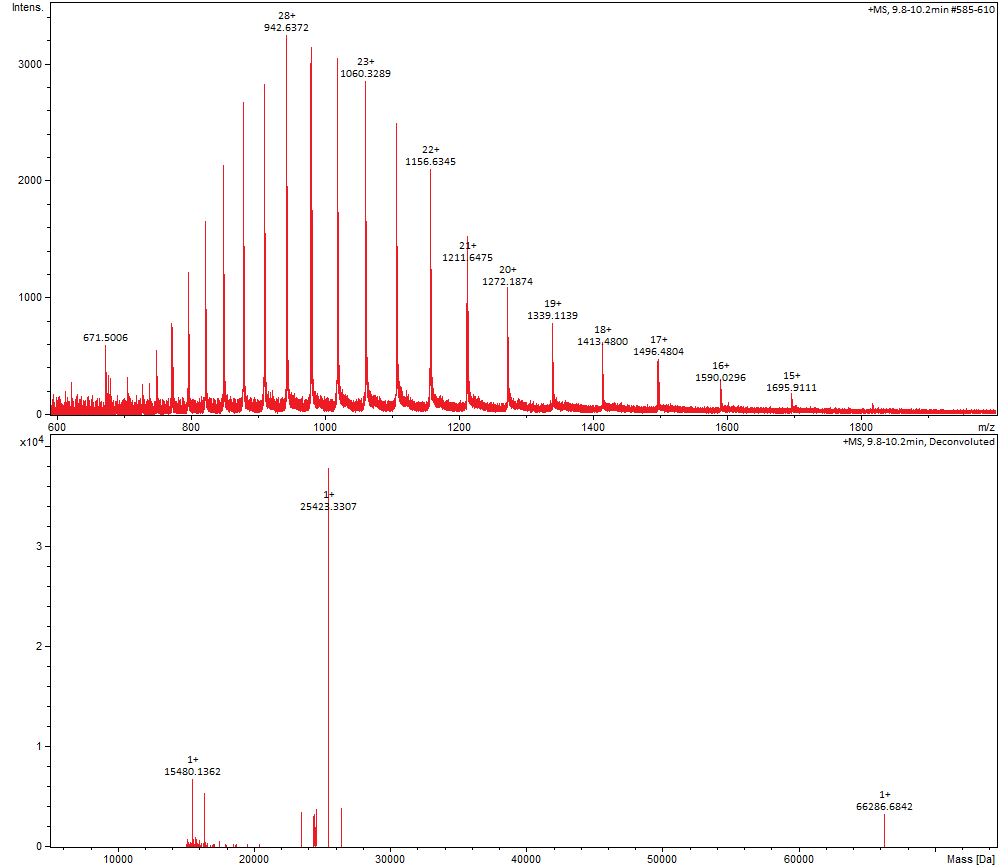


**Supplementary Figure 2.** Exemplary ESI-MS Spectra. Original trace data before deconvolution and deconvoluted spectra for ncAA **35**, biotinylated lysine ncAA, in mTFP1_TAG128_.

**Supplementary Figure 3.** Incorporation of ncAA **35** into mTFP1_TAG128_. Top: Coomassie stained SDS-PAGE of purified mTFP1wt, negative control of mTFP1_TAG128_ and mTFP1_ncAA_**_35_**; bottom: Fluorescence measurement of Alexa Fluor® 532 streptavidin conjugated to mTFP1_ncAA_**_35_**. Marker bands indicate 36 and 55 kDa.

**Supplementary Figure 4.** Fluorescence intensity mTFP1_TAG128_ co-expressed with HpRS or PylRS (Y271A and Y349F) in the presence of 1 mM **15**-**17**. Experiment was performed in triplicates.


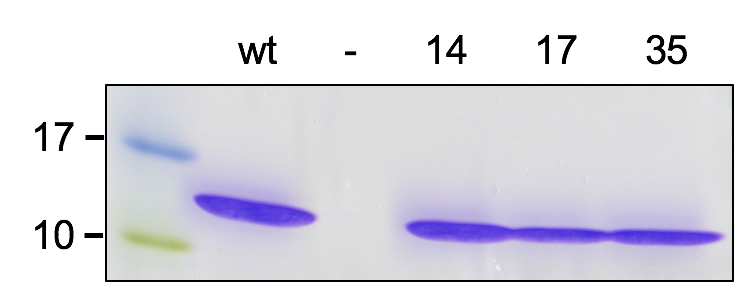


**Supplementary Figure 5.** Coomassie-stained SDS-PAGE of purified GFP-nanobody wt and GFP-nanobody containing ncAA **14**, **17**, or **35**.

**S2. Synthesis**

**S2.1 General Procedure**

N,N-Disuccinimidyl carbonate (DSC) (0.613 g, 1.2 eq.) and pyridine (0.28 ml, 3.0 eq.) were added to a solution of alcohol (2.0 mmol) in anhydrous dimethylformamide (DMF) (3 mL). The mixture was stirred at 40 °C for 15 h until the alcohol was completely activated, as observed by the thin-layer chromatography control. The mixture was cooled to room temperature and Boc-*L*-lysine-OtBu (Novabiochem, 2 mmol) was added at a rate that kept the reaction temperature below 30 °C. Then, the mixture was stirred overnight at room temperature. After the carbamate was formed (TLC control), H_2_O (10 mL) and EtOAc (10 mL) were added to the mixture. The organic layer was separated, and the aqueous layer was extracted with EtOAc (5 mL). The organic layer was subsequently washed with 1 N HCl, H_2_O, and brine, then dried on MgSO_4_. To protect the ncAAs as an oil, the organic solution was concentrated and column chromatography (n-hexane : EtOAC = 1 : 1) was performed. Then, a 1:1 mixture of TFA : CH_2_Cl_2_ was used for deprotection. After completing deprotection (confirmed by TLC), all volatiles were removed *in vacuo* and any residue was dissolved in methanol. Cold diethyl ether was added to precipitate the pure ncAA, which was filtered out and dried *in vacuo*.

**S2.1.1 Synthesis of ncAA 2**

Boc-ncAA-OtBu **2**

^1^H-NMR (CDCl_3_, 400 MHz): δ = 1.23-1.27 (m, 2H), 1.30 (s, 9H), 1.32 (s, 9H), 1.35-1.63 (m, 4H), 2.19-2.24 (m, 2H), 2.99-3.04 (m, 2H), 3.93-4.00 (m, 3H), 4.90-4.98 (m, 2H), 5.16 (br, 2H), 5.59-5.69 (m, 1H).

^13^C-NMR (CDCl_3,_ 100 MHz): δ = 171.86, 156.62, 155.39, 134.14, 116.82, 81.50, 79.31, 63.56, 53.70, 46.72, 33.38, 27.83, 22.24.

ncAA **2**

^1^H-NMR (D_2_O, 400 MHz): δ = 1.42-158 (m, 4H), 1.89 (m, 2H), 2.34-2.39 (dd, *J* = 8.0, 4.0 Hz, 1H), 3.13 (t, *J* = 6.0 Hz), 3.89 (t, *J* = 7.5 Hz), 4.06 (m, 2H), 5.07 (m, 2H), 5.84 (m, 1H).

^13^C-NMR (D_2_O, 100 MHz): δ = 174.25, 158.80, 134.80, 117.26, 64.35, 54.45, 39.88, 29.98, 21.56.

HRMS: *m*/*z* calcd for C_20_H_36_N_2_O_6_ [M + Na]^+^: 400.2573; found:

**S2.1.2 Synthesis of ncAA 3**

Boc-ncAA-OtBu **3**

^1^H-NMR (CDCl_3_, 400 MHz): δ = 1.30 (s, 9H), 1.32 (s, 9H), 1.34-1.63 (m, 8H), 1.96 (d, 2H, *J* = 4.0 Hz), 3,0 (br, 2H), 4.82 (d, *J* = 8.0 Hz), 4.87 (d, *J* = 16.0 Hz), 5.16 (br, 2H), 5.19 (m, 2H), 5.59-5.69 (m, 1H).

^13^C-NMR (CDCl_3,_ 100 MHz): δ = 171.83, 156.71, 155.37, 137.44, 114.93, 81.42, 79.22, 63.84, 53.68, 40.36, 29.85, 28.17, 27.81, 22.24.

ncAA **3**

^1^H-NMR (D_2_O, 400 MHz): δ = 0.99-1.21 (m, 4H), 1.22-1.25 (m, 2H), 1.43 (m, 2H), 1.53 (q, *J* = 16.0 Hz, 1H), 3.59 (m, 3H), 4.55 (d, *J* = 8.0 Hz), 4.60 (d, *J* = 16.0 Hz), 5.37-5.42 (m, 1H).

^13^C-NMR (D_2_O, 100 MHz): ^13^C-NMR (CDCl_3,_ 100 MHz): δ = 171.46, 158.41, 137.99, 114.45, 64.54, 52.39, 39.62, 29.07, 28.17, 27.14, 21.18.

**S2.1.3 Synthesis of ncAA 4**

Boc-ncAA-OtBu **4**

^1^H-NMR (CDCl_3_, 400 MHz): δ = 1.21 (s, 9H), 1.24 (s, 9H), 1.32-1.40 (m, 10H), 1.85 (dd, 2H, *J* = 10.0 Hz), 2.91 (br, 2H), 3.81(t, 2H, *J* = 4.0 Hz), 3.90 (m, 1H), 4.73 (d, *J* = 12.0 Hz), 4.77 (d, *J* = 20.0 Hz), 5.23-5.35 (m, 2H), 5.55 (m, 1H).

^13^C-NMR (CDCl_3,_ 100 MHz): δ = 171.79, 156.74, 155.33, 138.07, 114.53, 81.22, 79.02, 64.16, 53.69, 40.23, 33.09, 32.01, 29.24, 28.31, 28.10, 27.72, 24.90, 22.21.

ncAA **4**

^1^H-NMR (D_2_O, 400 MHz): δ = 0.63-0.83 (m, 8H), 1.14-1.25 (m, 4H), 2.32 (m, 2H), 3.23 (m, 3H), 4.13 (d, 2H, *J* = 12.0 Hz), 4.77 (d, 2H, *J* = 20.0 Hz), 5.02 (m, 1H).

^13^C-NMR (D_2_O_,_ 100 MHz): δ = 170.75, 157.88, 137.79, 113.32, 64.53, 52.02, 30.02, 27.02, 23.87, 20.93.

**S2.1.4 Synthesis of ncAA 5**

Boc-ncAA-OtBu **5**

^1^H-NMR (CDCl_3_, 400 MHz): δ = 1.31 (s, 9H), 1.33 (s, 9H), 1.41-1.49 (m, 12H), 1.92 (dd, 2H, *J* = 6.0 Hz), 3.02 (br, 2H), 3.91(t, 2H, *J* = 6.0 Hz), 4.01 (br, 1H), 4.80 (d, *J* = 9.0 Hz), 4.86 (d, *J* = 16.0 Hz), 5.16 (br, 2H), 5.67 (m, 1H).

^13^C-NMR (CDCl_3,_ 100 MHz): δ = 171.86, 156.80, 155.38, 138.50, 114.34, 81.41, 79.28, 64.53, 53.68, 40.40, 33.48, 32.27, 29.35, 28.78, 28.38, 28.20, 27.84, 25.21, 22.25.

ncAA **5**

^1^H-NMR (D2O, 400 MHz): δ = 0.54-0.80 (m, 12H), 1.22 (m, 2H), 2.32 (m, 2H), 3.26 (m, 3H), 4.12 (d, 2H, *J* = 20.0 Hz), 4.20 (d, 2H, *J* = 16.0 Hz), 4.63 (m, 1H).

^13^C-NMR (D2O,100 MHz): δ = 171.05, 158.05, 138.67, 113.12, 64.79, 52.13, 39.31, 32.27,28.77, 27.77, 27.20, 23.98, 20.85.

**S2.1.5 Synthesis of ncAA 6**

Boc-ncAA-OtBu **6**

^1^H-NMR (CDCl_3_, 400 MHz): δ = 1.21 (s, 9H), 1.23 (s, 9H), 1.15-1.37 (m, 14H), 1.81 (dd, 2H, *J* = 6.0 Hz), 2.92 (br, 2H), 3.80 (M, 2H), 3.90 (m, 1H), 4.70 (d, *J* = 12.0 Hz), 4.75 (d, *J* = 16.0 Hz), 5.24-5.35 (m, 2H), 5.52-5.58 (m, 1H).

^13^C-NMR (CDCl_3,_ 100 MHz): δ = 171.79, 156.76, 155.33, 138.49, 114.12, 81.17, 78.98, 64.33, 53.68, 40.21, 33.42, 32.01, 29.24, 28.72, 28.52, 27.71, 25.60, 22.20.

ncAA **6**

^1^H-NMR (D2O, 400 MHz): δ = 0.26-0.68 (m, 14H), 1.15 (m, 2H), 2.24 (m, 2H), 3.15 (m, 3H), 4.03 (d, 2H, *J* = 12.0 Hz), 4.10 (d, 2H, *J* = 16.0 Hz), 4.91-4.97 (m, 1H).

^13^C-NMR (D2O, 100 MHz): δ = 170.86, 158.04, 138.63, 113.12, 65.13, 52.47, 39.11, 32.09, 28.77, 27.70, 27.30, 24.60, 20.83.

**S2.1.6 Synthesis of ncAA 7**

Boc-ncAA-OtBu **7**

^1^H-NMR (CDCl_3_, 400 MHz): δ = 1.18 (s, 9H), 1.24 (s, 9H), 1.12-1.37 (m, 16H), 1.83 (dd, 2H, *J* = 6.0 Hz), 2.96 (m, 2H), 3.82 (m, 2H), 3.93 (m, 1H), 4.72 (d, *J* = 12.0 Hz), 4.78 (d, *J* = 16.0 Hz), 5.25-5.30 (m, 2H), 5.55-5.60 (m, 1H).

^13^C-NMR (CDCl_3,_ 100 MHz): δ = 171.80, 156.77, 155.34, 138.63, 114.07, 81.23, 79.03, 64.40, 53.68, 40.25, 33.51, 32.54, 29.26, 28.92, 28.76, 28.58, 28.12, 27.74, 25.61, 22.21.

ncAA **7**

^1^H-NMR (D2O, 400 MHz): δ = 0.60-0.88 (m, 14H), 1.21 (m, 4H), 2.40 (m, 2H), 3.29 (m, 3H), 3.34 (t, 1H, *J* = 8.0 Hz), 4.19 (d, 2H, *J* = 8.0 Hz), 4.26 (d, 2H, *J* = 20.0 Hz), 5.04-5.10 (m, 1H).

^13^C-NMR (D2O, 100 MHz): δ = 171.05, 158.04, 138.19, 113.11, 64.80, 52.21, 39.46, 32.83, 28.83, 28.24, 28.05, 28.00, 27.94, 27.86, 24.84, 21.07.

**S2.1.7 Synthesis of ncAA 8**

Boc-ncAA-OtBu **8**

^1^H-NMR (CDCl_3_, 400 MHz): δ = 1.29 (s, 9H), 1.34 (s, 9H), 1.15-1.37 (m, 18H), 1.88 (dd, 2H, *J* = 6.0 Hz), 3.00 (m, 2H), 3.87 (m, 2H), 3.97 (m, 1H), 4.76 (d, *J* = 8.0 Hz), 4.82 (d, *J* = 16.0 Hz), 5.22 (m, 2H), 5.58-5.68 (m, 1H).

^13^C-NMR (CDCl_3,_ 100 MHz): δ = 171.85, 156.84, 155.39, 138.77, 114.05, 81.38, 79.20, 64.55, 62.31, 53.68, 40.32, 33.59, 29.31, 29.29, 29.20, 29.08, 28.91, 28.85, 28.70, 28.16, 27.79, 25.69, 22.23.

ncAA **8**

^1^H-NMR (D2O, 400 MHz): δ = 0.7-1.06 (m, 16H), 1.42-1.49 (m, 4H), 2.56 (m, 2H), 3.45 (m, 2H), 3.51 (t, 1H, *J* = 8.0 Hz), 4.35 (d, 2H, *J* = 8.0 Hz), 4.41 (d, 2H, *J* = 16.0 Hz), 5.17-5.27 (m, 1H).

^13^C-NMR (D2O, 100 MHz): δ = 171.24, 158.04, 138.12, 113.37, 65.02, 52.23, 39.86, 33.15, 28.79, 28.66, 28.49, 28.31, 28.20, 25.01, 21.26.

**S2.1.8 Synthesis of ncAA 9**

Boc-ncAA-OtBu **9**

^1^H-NMR (CDCl_3_, 400 MHz): δ = 1.21 (s, 9H), 1.28 (s, 9H), 1.52 (m, 20H), 1.96 (dd, 2H, *J* = 6.0 Hz), 3.08 (m, 2H), 3.95 (m, 2H), 4.07 (m, 1H), 4.85 (d, *J* = 8.0 Hz), 4.86 (d, *J* = 16.0 Hz), 5.15 (m, 1H), 5.76-5.78 (m, 1H).

^13^C-NMR (CDCl_3,_ 100 MHz): δ = 171.89, 156.83, 155.41, 138.99, 114.07, 81.61, 79.42, 64.72, 53.68, 40.47, 33.59, 29.60, 29.39, 29.31, 29.19, 29.00, 28.81, 28.25, 28.70, 27.90, 27.79, 25.69, 21.95.

ncAA **9**

^1^H-NMR (D2O, 400 MHz): δ = -0.18-0.4 (m, 18H), 0.68 (m, 4H), 2.56 (m, 2H), 1.82 (m, 2H), 2.71 (m, 3H), 3.57 (d, 2H, *J* = 12.0 Hz), 3.65 (d, 2H, *J* = 16.0 Hz), 5.17-5.27 (m, 1H).

^13^C-NMR (D2O, 100 MHz): δ = 170.34, 157.91, 138.93, 111.94, 64.99, 51.84, 38.79, 31.94, 28.17, 27.42, 27.35, 27.22, 27.12, 27.01, 26.84, 23.87, 20.34.

**S2.1.9 Synthesis of ncAA 10**

Boc-ncAA-OtBu **10**

^1^H-NMR (CDCl_3_, 400 MHz): δ = 1.31 (s, 9H), 1.33 (s, 9H), 1.57 (m, 6H), 2.47 (m, 2H), 3.20 (m, 2H), 4.15 (m, 1H), 4.68 (br, 2H), 4.97 (br, 1H), 5.11 (br, 1H).

^13^C-NMR (CDCl_3,_ 100 MHz): δ = 171.87, 156.18, 155.41, 81.67, 80.34, 79.49, 69.55, 62.36 53.67, 40.55, 33.59, 29.60, 28.27, 27.92, 27.81, 22.27, 19.32.

ncAA **10**

^1^H-NMR (D_2_O, 400 MHz): δ = 1.31 (m, 2H), 1.45 (m, 2H), 1.78 (m, 2H), 2.78 (m, 1H), 3.05 (t, 2H, *J* = 6.0 Hz), 3.67(t, 2H, *J* = 6.0 Hz), 4.56 (s, 2H).

^13^C-NMR (D_2_O_,_ 100 MHz): δ = 174.34, 157.68, 78.81, 75.53, 54.72, 52.25, 40.09, 30.01, 28.16, 21.53.

**S2.1.10 Synthesis of ncAA 11**

Boc-ncAA-OtBu **11**

^1^H-NMR (CDCl_3_, 400 MHz): δ = 1.31 (s, 9H), 1.33 (s, 9H), 1.27-1.53 (m, 6H), 1.94 (t, 1H, *J* = 8.0 Hz), 2.43 (t, 2H, *J* = 6.0 Hz), 3.08 (m, 2H), 4.07 (m, 3H), 5.09 (br, 2H).

^13^C-NMR (CDCl_3,_ 100 MHz): δ = 171.87, 156.18, 155.41, 81.67, 80.34, 79.49, 69.55, 62.36 53.67, 40.55, 33.59, 29.60, 28.27, 27.92, 27.81, 22.27, 19.32.

ncAA**11**

^1^H-NMR (D_2_O, 400 MHz): δ = 1.26-1.39 (m, 4H), 1.68-1.75 (m, 2H), 2.28 (m, 1H), 2.43 (m, 2H), 3.02 (m, 2H), 3.63 (m, 1H), 4.03 (m, 2H).

^13^C-NMR (D_2_O_,_ 100 MHz): δ = 174.59, 158.36, 82.02, 70.79, 62.68, 54.60, 40.14, 30.04, 28.56, 21.55, 18.66.

**S2.1.11 Synthesis of ncAA 12**

Boc-ncAA-OtBu **12**

^1^H-NMR (CDCl_3_, 400 MHz): δ = 1.31 (s, 9H), 1.33 (s, 9H), 1.41-1.59 (m, 6H), 1.56-1.59 (m, 2H), 1.68 (m, 1H), 2.21 (t, 2H, *J* = 6.0 Hz), 3.10 (t, 2H, *J* = 6.0 Hz), 4.09 (m, 3H), 4.92-5.09 (br, 2H).

^13^C-NMR (CDCl_3,_ 100 MHz): δ = 171.87, 156.53, 155.43, 83.15, 81.73, 79.54, 68.89, 63.20, 53.66, 40.63, 32.53, 28.28, 27.94, 22.30, 15.10.

ncAA **12**

^1^H-NMR (D_2_O, 400 MHz): δ = 1.28-1.41 (m, 4H), 1.70-1.74 (m, 4H), 2.22 (m, 2H), 2.23 (m, 1H), 3.01 (m, 2H), 3.63 (t, 2H, *J* = 6.0 Hz), 4.04 (t, 2H, *J* = 6.0 Hz).

^13^C-NMR (D_2_O_,_ 100 MHz): δ = 174.25, 158.77, 84.88, 69.55, 64.09, 54.50, 39.87, 30.00, 28.52, 27.21, 21.56, 14.32.

**S2.1.12 Synthesis of ncAA 13**

Boc-ncAA-OtBu **13**

^1^H-NMR (CDCl_3_, 400 MHz): δ = 1.19 (s, 9H), 1.21 (s, 9H), 1.26-1.35 (m, 10H), 1.78 (m, 1H), 1.96 (m, 2H), 2.90 (m, 2H), 3.81(m, 2H), 3.87 (m, 1H), 5.23 (m, 1H), 5.39 (br, 1H).

^13^C-NMR (CDCl_3,_ 100 MHz): δ = 171.77, 156.63, 155.31, 83.58, 81.18, 78.99, 68.81, 63.68, 53.68, 40.22, 28.09, 27.90, 27.71, 24.64, 22.21, 17.79.

ncAA **13**

^1^H-NMR (D_2_O, 400 MHz): δ = 0.98-1.24 (m, 8H), 1.41 (m, 2H), 1.51 (m, 2H), 1.75 (t, 2H, *J* = 4.0 Hz), 1.77 (m, 1H), 2.66 (t, 2H, *J* = 6.0 Hz), 3.60 (m, 2H).

^13^C-NMR (D_2_O_,_ 100 MHz): δ = 171.45, 158.44, 85.55, 68.87, 64.68, 52.38, 39.59, 28.10, 27.05, 23.93, 21.15, 16.88.

**S2.1.13 Synthesis of ncAA 14**

Boc-ncAA-OtBu **14**

^1^H-NMR (CDCl_3_, 400 MHz): δ = 1.29 (s, 9H), 1.32 (s, 9H), 1.18-1.47 (m, 6H), 1.64 (m, 1H), 3.03 (dd, 2H, *J* = 8.0 Hz), 4.01 (m, 3H), 5.18 (m, 2H).

^13^C-NMR (CDCl_3,_ 100 MHz): δ = 171.81, 156.38, 155.41, 81.53, 79.33, 61.39, 60.20, 53.68, 48.10, 40.47, 28.47, 28.18, 27.82, 22.27, 20.83.

ncAA **14**

^1^H-NMR (D_2_O, 400 MHz): δ = 1.42-1.60 (m, 4H), 1.94 (m, 4H), 3.17 (m, 2H), 3.46 (m, 2H), 3.93 (m, 1H), 4.17 (m, 2H).

^13^C-NMR (D_2_O_,_ 100 MHz): δ = 173.60, 159.12, 65.98, 62.64, 53.88, 48.02, 39.89, 27.65, 27.01, 21.50.

**S2.1.14 Synthesis of ncAA 18**

Boc-ncAA-OtBu **18**

^1^H-NMR (CDCl_3_, 400 MHz): δ = 1.28 (s, 9H), 1.30 (s, 9H), 1.38 (m, 6H), 1.78 (m, 1H), 3.04 (dd, 2H, *J* = 6.0 Hz), 3.98 (m, 1H), 4.30 (dd, 2H, *J* = 8.0 Hz), 5.24 (m, 1H), 5.89 (m, 1H).

^13^C-NMR (CDCl_3,_ 100 MHz): δ = 171.84, 155.47, 154.48, 121.72, 124.47, 81.49, 79.30, 60.24, 60.15, 59.88, 53.65, 40.66, 28.89, 28.01, 27.65, 27.39, 22.12.

ncAA **18**

^1^H-NMR (D_2_O, 400 MHz): δ = 0.65-0.77 (m, 4H), 1.11-1.20 (m, 2H), 2.36 (t, 2H, *J* = 4.0 Hz), 3.25 (t, 2H, *J* = 6.0 Hz), 3.71 (q, 2H, *J* = 8.0 Hz).

^13^C-NMR (D_2_O_,_ 100 MHz): δ = 170.87, 155.70, 52.08, 39.40, 28.65, 27.47, 20.51.

**S2.1.15 Synthesis of ncAA 19**

Boc-ncAA-OtBu **19**

^1^H-NMR (CDCl_3_, 400 MHz): δ = 1.31 (s, 9H), 1.33 (s, 9H), 1.38-1.65 (m, 8H), 3.08 (dd, 2H, *J* = 6.0 Hz), 4.03 (m, 1H), 4.45 (dd, 2H, *J* = 12.0 Hz), 5.20 (m, 1H), 5.76 (m, 1H).

^13^C-NMR (CDCl_3,_ 100 MHz): δ = 171.86, 155.48, 154.53, 81.57, 79.37, 60.24, 60.15, 59.88, 53.63, 40.76, 28.89, 28.01, 27.62, 27.39, 22.13.

ncAA **19**

^1^H-NMR (D_2_O, 400 MHz): δ = 0.65-0.77 (m, 4H), 1.11-1.20 (m, 2H), 2.36 (t, 2H, *J* = 4.0 Hz), 3.25 (t, 2H, *J* = 6.0 Hz), 3.71 (q, 2H, *J* = 8.0 Hz).

^13^C-NMR (D_2_O_,_ 100 MHz): δ = 170.87, 155.70, 52.08, 39.40, 28.65, 27.47, 20.51.

**S2.1.16 Synthesis of ncAA 20**

Boc-ncAA-OtBu **20**

^1^H-NMR (CDCl_3_, 400 MHz): δ = 1.31 (s, 9H), 1.33 (s, 9H), 1.38-1.65 (m, 8H), 3.08 (dd, 2H, *J* = 6.0 Hz), 4.03 (m, 1H), 4.45 (dd, 2H, *J* = 12.0 Hz), 5.20 (m, 1H), 5.76 (m, 1H).

^13^C-NMR (CDCl_3,_ 100 MHz): δ = 171.86, 155.48, 154.53, 81.57, 79.37, 60.24, 60.15, 59.88, 53.63, 40.76, 28.89, 28.01, 27.62, 27.39, 22.13.

ncAA **20**

^1^H-NMR (D_2_O, 400 MHz): δ = 0.81-0.94 (m, 4H), 1.33 (m, 2H), 2.52 (t, 2H, *J* = 8.0 Hz), 3.42 (m, 2H), 3.71 (t, 2H, *J* = 12.0 Hz).

^13^C-NMR (D_2_O_,_ 100 MHz): δ = 171.47, 155.92, 51.77, 39.43, 28.89, 27.12, 20.76.

**S2.1.17 Synthesis of ncAA 21**

Boc-ncAA-OtBu **21**

^1^H-NMR (CDCl_3_, 400 MHz): δ = 1.37 (s, 9H), 1.39 (s, 9H), 1.31-1.56 (m, 6H), 2.27 (M, 2H), 3.11 (m, 2H), 4.03 (m, 1H), 4.30 (m, 2H), 5.17 (m, 1H), 5.24 (m, 1H).

^13^C-NMR (CDCl_3,_ 100 MHz): δ = 171.87, 155.88, 154.53, 81.67, 79.48, 56.42, 53.64, 40.56, 28.89, 28.01, 27.74, 22.23.

ncAA **21**

^1^H-NMR (D_2_O, 400 MHz): δ = 0.60-0.73 (m, 4H), 1.11 (m, 2H), 1.63 (m, 2H), 2.29 (t, 2H, *J* = 6.0 Hz), 3.20 (m, 1H), 3.50 (t, 2H, *J* = 8.0 Hz).

^13^C-NMR (D_2_O_,_ 100 MHz): δ = 171.46, 157.06, 56.67, 51.77, 39.46, 28.67, 27.10, 20.81.

**S2.1.18 Synthesis of ncAA 22**

Boc-ncAA-OtBu **22**

^1^H-NMR (CDCl_3_, 400 MHz): δ = 1.26 (s, 9H), 1.28 (s, 9H), 1.38 (m, 6H), 2.24-2.35 (m, 2H), 3.00 (m, 2H), 3.98 (m, 1H), 4.18 (m, 2H), 5.28 (m, 1H), 5.58 (m, 1H).

^13^C-NMR (CDCl_3,_ 100 MHz): δ = 171.88, 155.91, 155.50, 81.66, 79.48, 56.44, 53.64, 40.55, 32.41, 30.96, 30.74, 30.53, 29.12, 28.04, 27.70, 22.23.

ncAA **22**

^1^H-NMR (D_2_O, 400 MHz): δ = 0.36-0.45 (m, 4H), 0.85-0.92 (m, 2H), 1.36 (m, 2H), 2.05 (m, 2H), 2.96 (m, 1H), 3.26 (m, 2H).

^13^C-NMR (D_2_O_,_ 100 MHz): δ = 170.69, 156.87, 56.33, 51.78, 39.08, 28.41, 27.11, 20.46.

**S2.1.19 Synthesis of ncAA 23**

Boc-ncAA-OtBu **23**

^1^H-NMR (CDCl_3_, 400 MHz): δ = 1.27 (s, 9H), 1.29 (s, 9H), 1.35-1.60 (m, 6H), 1.97 (dd, 2H, *J* = 4.0 Hz), 3.01 (m, 3H), 4.03 (m, 2H), 5.21 (m, 1H), 5.47 (m, 1H).

^13^C-NMR (CDCl_3,_ 100 MHz): δ = 171.80, 155.95, 155.44, 81.41, 79.22, 60.50, 53.67, 40.40, 32.12, 27.95, 27.58, 22.19.

ncAA **23**

^1^H-NMR (D_2_O, 400 MHz): δ = 1.35-1.48 (m, 4H), 1.78 (m, 2H), 2.09 (m, 2H), 3.03 (t, 2H, *J* = 6.0 Hz), 3.95 03 (t, 2H, *J* = 8.0 Hz), 4.10 (m, 2H).

^13^C-NMR (D_2_O_,_ 100 MHz): δ = 172.17, 158.22, 66.97, 52.82, 39.78, 29.35, 28.31, 22.81, 21.38, 16.91.

**S2.2 Synthesis of ncAAs with Acid-Sensitive Carbamates (ncAAs 28 and 29)**

**S2.2.1 General Procedure**

**S2.2.2 Synthesis of the Nitrophenol-Carbamate Precursor (NC-ncAA)**

Alcohol (1.54 g, 10.0 mmol) and TEA (1.70 ml, 12 mmol, 1.2 eq) were dissolved in THF (40 ml) and dripped into a stirred solution of 4-nitrophenyl chloroformate (4-NCF, 6.04 g, 30 mmol, 3.0 eq) in THF (36 ml) over a period of 1 h at -10 °C. The reaction mixture was allowed to warm to room temperature and was stirred overnight. THF was removed *in vacuo*, and water was added to the residue. The mixture was extracted three times with EtOAc. The collected organic phase were dried on MgSO_4_ following by column chromatography (n-hexane : EtOAc = 9 : 1) to yield the desired yellow-oil product.

**S2.2.3 Synthesis of the Fmoc-Protected ncAA (Fmoc-ncAA)**

Fmoc-*L*-Lys-OH (Novabiochem, 0.69 g, 1.87 mmol, 1.2 eq.) was suspended under argon in anhydrous DMF (0.2 M, 8 ml) containing DIEA (0.24 g, 0.33 ml, 1.87 mmol, 1.2 eq.). To this white suspension, a clear solution of the nitrophenol-carbamate precursor (1.56 mmol, 1.0 eq.) in anhydrous DMF (0.2 M, 8 ml) was added dropwise under argon at room temperature over a period of 2 h. The reaction mixture was stirred for an additional 4 h at room temperature. H_2_O (50 ml) and EtOAc (150 ml) were added and the aqueous layer was adjusted to a pH range of 1–3 with 1 N HCl. The phases were separated and the aqueous layer was extracted with EtOAc (2 x 50 ml). The organic layers were washed with saturated NaCI solution (2 x 50 ml) and dried on Na_2_SO_4_. All volatiles were evaporated under reduced pressure and the crude product was purified by column chromatography (DCM : MeOH 95 : 5 v/v) to yield the Fmoc-protected ncAA as a white solid.

**S2.2.4 Deprotection of the Fmoc-ncAA**

The Fmoc-protected ncAA was dissolved in 20% piperidine in DMF (20 ml per mmol of ncAA) and stirred for 1 h at room temperature. All volatiles were removed under reduced pressure to yield the pure compound as a solid.

**S2.3.1 Synthesis of ncAA 28**

NC-ncAA **28**

^1^H-NMR (CDCl_3_, 400 MHz): δ = 1.63 (s, 3H), 1.70 (s, 3H), 1.83 (s, 3H), 2.15 (m, 4H), 4.78 (d, 2H, *J* = 4.0 Hz), 5.10 (m, 1H), 5.47 (t, 1H, *J* = 8.0 Hz), 7.40 (d, 2H, *J* = 12.0 Hz), 8.29 (d, 2H, *J* = 8.0 Hz).

Fmoc-ncAA **28**

^1^H-NMR (CDCl_3_, 400 MHz): δ = 1.62 (s, 3H), 1.70 (s, 3H), 1.76 (s, 3H), 1.44-1.91 (m, 6H), 2.09 (m, 2H), 3.16 (m, 2H), 4.41 (m, 2H), 4.59 (m, 2H), 5.11 (m, 2H), 5.35 (m, 1H), 5.97 (d, *J* = 4.0 Hz), 7.31 (t, 2H, *J* = 8.0 Hz), 7.39 (t, 2H, *J* = 6.0 Hz), 7.55-7.63 (m, 2H), 7.66 (d, *J* = 8.0 Hz).

^13^C-NMR (CDCl_3,_ 100 MHz): δ = 176.25, 176.03, 175.63, 171.51, 157.13, 156.44, 143.96, 143.78, 142.14, 141.34, 132.12, 127.13, 123.69, 120.00, 67.14, 60.57, 53.70, 47.21, 40.63, 32.20, 29.41, 26.72, 25.72, 22.38, 21.07, 20.79, 17.70, 14.22.

ncAA **28**

^1^H-NMR (D_2_O, 400 MHz): δ = 1.64 (s, 3H), 1.71 (s, 3H), 1.75 (s, 3H), 1.20-1.91 (m, 6H), 2.16 (m, 2H), 3.15 (m, 2H), 3.75 (m, 1H), 4.55 (m, 2H), 4.59 (m, 2H), 5.42 (m, 1H).

**S2.3.2 Synthesis of ncAA 29**

NC-ncAA **29**

^1^H-NMR (CDCl_3_, 400 MHz): δ = 1.62 (s, 3H), 1.70 (s, 3H), 1.78 (s, 3H), 2.12 (m, 4H), 4.82 (d, 2H, *J* = 8.0 Hz), 5.10 (m, 1H), 5.47 (t, 1H, *J* = 8.0 Hz), 7.40 (d, 2H, *J* = 8.0 Hz), 8.29 (d, 2H, *J* = 8.0 Hz).

Fmoc-ncAA **29**

^1^H-NMR (CDCl_3_, 400 MHz): δ = 1.61 (s, 3H), 1.64 (s, 3H), 1.69 (s, 3H), 1.43-1.91 (m, 6H), 2.07 (m, 2H), 3.20 (m, 2H), 4.22 (m, 1H), 4.40 (m, 2H), 4.66-4.82 (m, 2H), 5.09 (m, 2H), 5.33 (m, 1H), 7.29 (t, 2H, *J* = 6.0 Hz), 7.38 (t, 2H, *J* = 6.0 Hz), 7.61 (m, 2H), 7.76 (d, *J* = 8.0 Hz).

^13^C-NMR (CDCl_3,_ 100 MHz): δ = 176.03, 175.20, 157.13, 156.35, 144.01, 143.88, 142.58, 141.29, 131.79, 127.07, 123.76, 119.96, 67.23, 62.00, 53.88, 47.15, 39.54, 31.73, 26.31, 25.69, 22.56, 17.70, 16.47.

ncAA **29**

^1^H-NMR (D_2_O, 400 MHz): δ = 1.60 (s, 3H), 1.66 (s, 3H), 1.70 (s, 3H), 1.46-1.89 (m, 6H), 2.05 (m, 2H), 3.11 (m, 2H), 3.67 (m, 1H), 4.55 (m, 2H), 5.32 (m, 1H).
